# Plants acclimate to Photosystem I photoinhibition by readjusting the photosynthetic machinery

**DOI:** 10.1111/pce.14400

**Published:** 2022-08-16

**Authors:** Tapio Lempiäinen, Eevi Rintamäki, Eva‐Mari Aro, Mikko Tikkanen

**Affiliations:** ^1^ Molecular Plant Biology, Department of Life Technologies University of Turku Turku Finland

**Keywords:** chloroplast proton‐translocating ATPases, cytochrome b_6_f complex, electron transport, light‐harvesting protein complexes, phosphorylation, photosynthesis, Photosystem I protein complex, Photosystem II protein complex, thioredoxins, thylakoids

## Abstract

Photosynthetic light reactions require strict regulation under dynamic environmental conditions. Still, depending on environmental constraints, photoinhibition of Photosystem (PSII) or PSI occurs frequently. Repair of photodamaged PSI, in sharp contrast to that of PSII, is extremely slow and leads to a functional imbalance between the photosystems. Slow PSI recovery prompted us to take advantage of the PSI‐specific photoinhibition treatment and investigate whether the imbalance between functional PSII and PSI leads to acclimation of photosynthesis to PSI‐limited conditions, either by short‐term or long‐term acclimation mechanisms as tested immediately after the photoinhibition treatment or after 24 h recovery in growth conditions, respectively. Short‐term acclimation mechanisms were induced directly upon inhibition, including thylakoid protein phosphorylation that redirects excitation energy to PSI as well as changes in the feedback regulation of photosynthesis, which relaxed photosynthetic control and excitation energy quenching. Longer‐term acclimation comprised reprogramming of the stromal redox system and an increase in ATP synthase and Cytochrome b_6_f abundance. Acclimation to PSI‐limited conditions restored the CO_2_ assimilation capacity of plants without major PSI repair. Response to PSI inhibition demonstrates that plants efficiently acclimate to changes occurring in the photosynthetic apparatus, which is likely a crucial component in plant acclimation to adverse environmental conditions.

## 1 INTRODUCTION

Photosynthesis utilizes light to assimilate CO_2_ into organic compounds. Photons are transduced to chemical energy in Photosystem II (PSII) and Photosystem I (PSI) reaction centres, which eject electrons to the linear electron transfer chain (LET) generating NADPH. Concomitant proton pumping to the thylakoid lumen establishes a proton motive force (pmf) that is utilized by ATP synthase to produce ATP from Pi and ADP. This energy transduction requires the concerted function of the four membrane‐embedded protein complexes, PSII, PSI, cytochrome b_6_f complex (Cyt b_6_f), and ATP synthase. Photosystems and Cyt b_6_f are connected by mobile electron carriers, membrane soluble plastoquinone (PQ), and lumenal plastocyanin (PC), whereas two soluble proteins, ferredoxin (Fd) and ferredoxin NADP^+^ oxidoreductase (FNR) on the stromal side of PSI finalize the reduction of NADP^+^ to NADPH. Most of the ATP and reducing power generated in LET are used to reduce CO_2_ to triose phosphates and to regenerate ribulose 1,5‐bisphosphate in the Calvin–Benson–Bassham (CBB) cycle. Other major sinks for reducing power in chloroplasts include photorespiration, antioxidant system, nitrogen assimilation (NA) and, to a smaller extent, also sulfur assimilation and lipid biosynthesis, all depending on environmental conditions (Asada, 1999; Foyer et al., 2009; Walker et al., 2020). In addition to these stromal reaction pathways, a part of the reducing power is exported from chloroplast through the malate valve (Selinski & Scheibe, 2018) to be utilized in other cellular compartments (Shameer et al., 2019). The distribution of reducing power between these different sinks needs to be regulated according to the metabolic state of the cell, predominantly occurring via the thioredoxin (Trx) system (Geigenberger et al., 2017; Schürmann & Buchanan, 2008).

Prompt and accurate regulation of photosynthetic light reactions is crucial, as the imbalance between PSII and PSI, as well as that between the light reactions and stromal sinks, leads to the formation of harmful reactive oxygen species (ROS) (Asada, 2006). Despite multilayered antioxidant systems scavenging ROS in chloroplasts, both PSII and PSI are prone to oxidative damage under harsh environmental conditions. The control of ROS production in LET is important not only to avoid uncontrolled oxidative damage but also to allow site‐specific ROS production in LET as an important secondary messenger for regulation of gene expression and promotion of plant long‐term acclimation to changed environmental conditions (Chan et al., 2016; de Souza et al., 2017; Farmer & Mueller, 2013; Fitzpatrick et al., 2022).

Photodamage and inhibition of PSI under conditions challenging the capacity of the regulatory and scavenging systems have been known to exist already for decades, particularly in chilling sensitive plants (Havaux & Davaud, 1994; Terashima et al., 1994). Nevertheless, the demonstration of PSI photoinhibition in *Arabidopsis thaliana* proton gradient regulation 5 (PGR5) mutant in natural fluctuating light conditions (Suorsa et al., 2012) boosted PSI photoinhibition research. In sharp contrast to the fast repair mechanisms of PSII upon photoinhibition (Aro et al., 1993; Nishiyama et al., 2011), PSI repair is known to be extremely slow (Kudoh & Sonoike, 2002) and to require the synthesis and assembly of the entire PSI complex. Depending on environmental conditions, the complete repair takes from days to weeks and during this time the photosynthetic light reactions can become limited by PSI (Lima‐Melo et al., 2019; Zhang & Scheller, 2004; Zhang et al., 2011; Zivcak et al., 2015). Thus, it is likely that plants mitigate the consequences of PSI inhibition by acclimation to the PSI complex deficiency.

**Figure 1:**
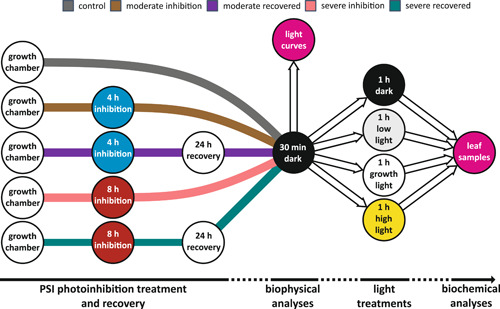
Experimental setup to investigate the recovery of *Arabidopsis* from Photosystem I (PSI) photoinhibition. For biophysical analyses, plants were PSI photoinhibited for 4 h (moderate inhibition) or 8 h (severe inhibition) after which half of the plants were returned to growth conditions to recover for 24 h. Control plants were taken directly from growth conditions. Five differently treated plant groups were dark acclimated for at least 30 min before light curves were recorded to investigate the function and regulation of photosynthetic light reactions, ATPase and CO_2_ assimilation. For biochemical analyses, another set of dark acclimated plants was divided into four groups, which were treated for 1 h in darkness (0 µmol photons m^−2^ s^−1^), low light (35 µmol photons m^−2^ s^−1^), growth light (165 µmol photons m^−2^ s^−1^), or high light (635 µmol photons m^−2^ s^−1^). After these light treatments, leaf samples were collected for thylakoid isolation and redox labelling of proteins, to study the regulation of photosynthesis at the protein level. [Color figure can be viewed at wileyonlinelibrary.com]

To disclose putative mechanisms that drive the acclimation of plants to PSI deficiency, without being hampered by concomitant low‐temperature stress, we subjected wild‐type *Arabidopsis* plants to moderate and severe PSI photoinhibition, using specific light treatments at normal growth temperature (Tikkanen & Grebe, 2018) to induce 60% and 85% PSI photoinhibition. The five sets of plants, (i) untreated, (ii) 60% PSI photoinhibited, (iii) 85% PSI photoinhibited, as well as (iv) 60% PSI photoinhibited and subsequently ‘recovered’ for 24 h in growth conditions, and (v) 85% PSI photoinhibited and subsequently ‘recovered’ for 24 h in growth conditions, were subjected to analyses of their major photosynthetic complexes, the function and regulation of photosynthetic light reactions, ATP synthase and carbon assimilation. These measurements were further complemented by analyses of the redox regulation of photosynthetic enzymes, as well as the phosphorylation and supercomplex formation of thylakoid proteins and complexes in different light conditions. The experimental setup is outlined in Figure 1.

## 2 MATERIALS AND METHODS

### 2.1 Growth conditions and light treatments

Wild‐type Columbia ecotype *A. thaliana* plants were grown in an 8 h photoperiod in constant moderate white light (165 µmol photons m^−2^ s^−1^) with POWERSTAR HQI‐T 400W/D metal halide lamps (OSRAM GmbH) as light source at 25°C and 60% relative humidity. The plants used in the experiments were 5–6 weeks old. The sequence of the plant treatments used in the experiments is depicted in Figure 1.

Whole plants were treated with a specific fluctuating light regime to induce PSI photoinhibition (Tikkanen & Grebe, 2018). The light treatment is described in Table 1.

**Table 1:** The fluctuating light regime used for PSI photoinhibition

Light quality	Light intensity (µmol photons m^−2^ s^−1^)	Duration (s)	Effect on photosynthetic machinery
White	100	20	Balanced electron flow
Red	35	5	Preferential excitation of PSII, accumulation of electrons in ETC
White	3200	1	Over‐reduction of PSI electron acceptors
Red	35	5	Preferential excitation of PSII, accumulation of electrons in ETC
White	3200	1	Over‐reduction of PSI electron acceptors
Red	35	5	Preferential excitation of PSII, accumulation of electrons in ETC
White	3200	1	Over‐reduction of PSI electron acceptors

Abbreviations: ETC, Electron transfer chain; PSI, Photosystem I; PSII, Photosystem II.

Plants were treated for 4 and 8 h to induce moderate and severe PSI photoinhibition. For the recovery, plants were transferred to growth conditions for 24 h after the inhibition treatments. Control plants were taken directly from growth conditions.

Before the biophysical analyses and the light treatments preceding the biochemical analyses, plants were subjected to 30 min dark adaptation. After that, plants were either directly used in biophysical measurements or further treated for 1 h as follows: dark, low light (35 µmol photons m^−2^ s^−1^), growth light (165 µmol photons m^−2^ s^−1^) or high light (635 µmol photons m^−2^ s^−1^) illumination. Leaf samples were then directly used for biochemical analyses or frozen in liquid nitrogen and stored at −80°C.

### 2.2 Biophysical analyses

CO_2_ assimilation, chlorophyll a fluorescence, P700, Fd and PC redox states were recorded concurrently with GSF‐3000 infra‐red gas analyser which was connected with a 3010‐Dual gas exchange cuvette to Dual‐KLAS‐NIR (Heinz Walz GmbH). The used light intensities are described in Table 2. Actinic light was supplemented with 10% blue light to ensure proper stomatal regulation. Measurements were done after a minimum of 30 min dark acclimation.

**Table 2:** Light intensities used for measurement of CO_2_ assimilation, chlorophyll a fluorescence, P700 and PC redox state

Light condition	Light intensity (µmol photons m^−2^ s^−1^)	Duration (min)	Analysis
Dark	0	1	*F*_M_‐determination pulse at the beginning and saturating pulse at the end
Low light	35	5	Saturating pulse every 1 min
Growth light	165	5	Saturating pulse every 1 min
High light	635	5	Saturating pulse every 1 min
Saturating light	2000	2	Saturating pulse every 1 min
Dark	0	1	Saturating pulse at the end

Abbreviation: PC, plastocyanin.

CO_2_ assimilation was recorded every 10 s during the light curve. The gas flow rate was set to 400 µmol s^−1^, the cuvette temperature was kept at a constant 25°C and the concentration of CO_2_ and H_2_O were set to 400 and 18 000 p.p.m., respectively. Assimilation was calculated according to (von Caemmerer & Farquhar, 1981). Saturating pulse of 4000 µmol photons m^−2^ s^−1^ was given every minute to determine the fluorescence and absorbance parameters described below.

Chlorophyll a fluorescence was detected with pulse‐modulated 540 nm measuring light. P700, Fd and PC redox states were determined by deconvolution of pulse‐modulated dual‐wavelength 785–840, 810–870, 870–970 and 795–970 nm signals. Deconvolution was performed using differential model plots measured from control plants (Klughammer & Schreiber, 2016). PSI inhibition prevents the formation of P700^+^, which means that only the functional reaction centres contribute to the P700 signal in PSI photoinhibited plants. Therefore, we calculated the yields of PSI using the average of control *P*_M_ measured with the NIR MAX script (Klughammer & Schreiber, 2016) for all treatments, following the approach used previously (Zivcak et al., 2015). This approach assigns the damaged PSI reaction centres as acceptor side limited. The photochemical quantum yield of PSI (Φ_I_), donor side limitation (Φ_ND_) and acceptor side limitation (Φ_NA_) were calculated according to (Klughammer & Schreiber, 1994, 2008b). To determine the yields of functional PSI centres (denoted with F) Φ_I_^F^, Φ_ND_^F^ and Φ_NA_^F^, we used the maximal P700 oxidation (*P*_M_*) for each leaf sample, which eliminates the contribution of damaged PSI reactions centres to Φ_I_^F^, Φ_ND_^F^ and Φ_NA_^F^. Due to PSI photoinhibition‐induced low PSI to PSII ratio, the far‐red light was not able to properly oxidize electron transfer chain (ETC) and thus the maximal oxidation of P700 and PC (*P*_M_* and *P*C_M_*, respectively) were determined with a saturating pulse under saturating light conditions at the end part of the light curve, a condition where the photosynthetic control is likely able to keep them maximally oxidized. Steady‐state oxidation of PC was calculated analogously to Φ_ND_. The photochemical quantum yield of PSII (Φ_II_), the yield of regulated energy dissipation (Φ_NPQ_) and the yield of nonregulated energy dissipation (Φ_NO_) were calculated according to Genty (Genty et al., 1989; Klughammer & Schreiber, 2008a). Fd results were not analysed further since the signal is partly originating from PSI iron‐sulfur clusters (Klughammer & Schreiber, 2016), which get damaged in the photoinhibition treatment.

The distribution of reducing power (electrons) to carbon assimilation was calculated according to equation 1.

$${\text{Distribution}\;\text{of}\;\text{electrons}\;\text{to}\;\text{CO}}_{2}\;\text{assimilation}=\frac{4\times \;\text{Assimilation}}{\text{ETRII}}=\frac{4\times \;\text{Assimilation}}{\Phi_{\text{II}}\times 0.5\times 0.84\times \;\text{PPDF}}$$

Equation 1: Distribution of reducing power to CO_2_ assimilation

Electrochromic shift (ECS) was recorded with Dual‐PAM‐100 equipped with P515/535 module (Heinz Walz GmbH). The used light intensities are described in Table 3. The electrochromic shift was determined by the difference between 515 and 550 nm signals (Schreiber & Klughammer, 2008). Dark interval of 250 ms was applied every minute to quantify the thylakoid proton conductivity (g_H+_) and the pmf (ECS_t_). The g_H+_ parameter was calculated as the inverse of the time constant of the first‐order exponential fit to the decay of the ECS signal during the dark interval (Kanazawa & Kramer, 2002). ECS_t_ was calculated as a difference between the ECS in the light before the dark interval and the ECS dark baseline calculated from the first‐order exponential fit. ECS_t_ was normalized to the chlorophyll content of leaf discs (10 mm in diameter) cut from leaves after the measurements. Chlorophyll content and chlorophyll a/b ratio were determined from dimethylformamide (DMF) leaf extracts (Inskeep & Bloom, 1985).

**Table 3:** Light intensities used for electrochromic shift measurements

Light condition	Light intensity (µmol photons m^−2^ s^−1^)	Duration (min)	Analysis
Dark	0	1	Single turnover pulse at the beginning
Low light	35	5	Dark interval every 1 min
Growth light	165	5	Dark interval every 1 min
High light	635	5	Dark interval every 1 min

### 2.3 Biochemical analyses

Western blot analysis was performed as described in Rantala et al. (2020), with primary antibodies raised against the following proteins: PsaB, AtpF, PetA (Agrisera product numbers: AS10 695, AS10 1604 and AS20 4377, respectively) and PsbA (Kettunen et al., 1996). Infra‐red dye‐labelled secondary antibody (IRDye® 800CW Goat anti‐Rabbit IgG Secondary Antibody [1:20 000 in 1% milk/TTBS, Li‐Cor]) was used in protein immune detection with Odyssey CLx imager (Li‐Cor). The antibody signal was quantified with the Image Studio programme (Li‐Cor). The relative amounts of proteins were interpolated from the linear regression of signals of the control dilution series.

Thylakoid proteins were separated with sodium dodecyl‐sulfate polyacrylamide gel electrophoresis (SDS‐PAGE) gel containing 12% acrylamide and 6 M urea. ProQ and Sypro Ruby staining of separated thylakoidal proteins were performed according to manufacturer's instructions (Invitrogen). Gels were imaged with Perkin Elmer Geliance 1000 using Cy3 filter for ProQ and UV‐filter for Sypro‐dyed gels.

77 K fluorescence measurements were performed with a chlorophyll concentration of 10 µg/ml. Thylakoids were excited by 480 nm light and fluorescence was detected with Ocean Optics S2000 spectrophotometer. Spectra were normalized to 685 nm peak and the ratio between 685 and 735 nm peaks was calculated to illustrate the distribution of excitation energy between PSII and PSI.

Blue native gel electrophoresis was performed as described in (Järvi et al., 2011). Isolated thylakoids were suspended into ice‐cold 25BTH20G buffer (25 mM BisTris‐HCl pH 7.0, 20% [v/v] glycerol and 0.25 mg/ml Pefabloc). Resuspended thylakoids were solubilized with an equal volume of 2% detergent solution (β‐*D*‐dodecyl maltoside [β‐DM] or digitonin) in 25BTH20G buffer. β‐DM samples were solubilized for 5 min on ice and digitonin samples were solubilized with gentle mixing for 8 min at room temperature. Insoluble material was removed by centrifugation at 16 000*g* for 20 min at 4°C and 1/10 volume of loading buffer (100 mM BisTris‐HCl pH 7.0, 0.5 M aminocaproic acid, 30% [w/v] sucrose and 50 mg/ml Serva Blue G) was added to the supernatant. Solubilized thylakoid protein complexes were separated with 3%–12% acrylamide gradient gels.

Protein redox labelling followed the protocol (Peled‐Zehavi et al., 2010). Leaves frozen in liquid nitrogen were homogenized in 10% trichloroacetic acid and the proteins were precipitated with centrifugation. Precipitated proteins were first labelled with 50 mM *N*‐ethyl maleimide to block the protein thiols followed by the reduction of residual disulfide bridges in proteins with dithiothreitol and subsequent labelling of exposed thiols with 10 mM pegylated maleimide (5 kDa) as described in Nikkanen et al. (2016). For estimation of protein content in the samples, the labelled proteins were separated with sodium dodecyl‐sulfate polyacrylamide gel electrophoresis (SDS‐PAGE) gel containing 12% acrylamide and 6 M urea and the gel was stained with Sypro Ruby protein stain and imaged with Perkin Elmer Geliance 1000. The protein content of the samples was normalized to the amount of rubisco small subunit. An equal amount of proteins in labelled samples was separated with SDS‐PAGE gel with 5%–15% acrylamide gradient with 6 M urea and electroblotted to polyvinylidene difluoride membrane (Millipore). Fructose 1,6‐bisphosphatase (FBPase) was identified with a specific antibody (a kind gift from Dr M. Sahrawy), using an enhanced chemiluminescence detection kit (GE Healthcare) and Perkin Elmer Geliance 1000 imager.

## 3 RESULTS

### 3.1 Photoinhibition treatment is specific for PSI

**Figure 2:**
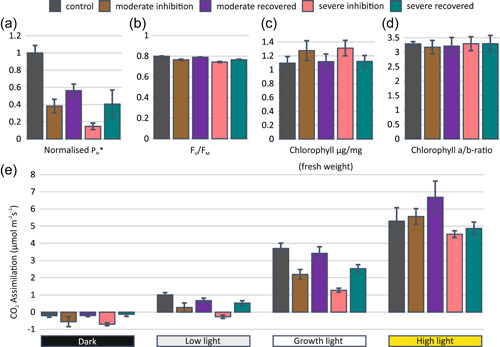
(a) Maximal oxidation of Photosystem I (PSI) (*P*_M_*) and (b) Photosystem II (PSII) maximal quantum efficiency (*F*_V_/*F*_M_) were measured with saturating pulse method from plants directly after the PSI photoinhibition treatment and after the plants had recovered for 24 h in growth conditions following the photoinhibition treatment. Control plants were taken directly from growth conditions. *P*_M_* was measured with saturating pulse at the end of the light curve during strong background illumination. *F*_V_/*F*_M_ was measured from darkness at the beginning of the light curve. Error bars show SDs among replicates (*n* ≥ 4). P_M_* values and SDs were normalized to the mean of control plants. (c) Chlorophyll content and (d) chlorophyll a/b ratio of leaves were determined from DMF extracts. Error bars show SDs among replicates (*n* ≥ 4). (e) Net CO_2_ assimilation rates of PSI photoinhibited and recovered plants were recorded from four different light intensities: dark, low light (35 µmol photons m^−2^ s^−1^), growth light (165 µmol photons m^−2^ s^−1^) and high light (635 µmol photons m^−2^ s^−1^). Assimilation was measured from plants directly after PSI inhibition and from plants that had recovered for 24 h in growth conditions after inhibition. Control plants were taken directly from growth conditions. All plants were dark acclimated for a minimum of 30 min before subsequent illumination at the indicated light intensities for 5 min. Error bars show SDs among replicates (*n* ≥ 4). [Color figure can be viewed at wileyonlinelibrary.com]

We first tested the specificity and effectivity of the PSI photoinhibition treatments by analysing the PSI maximal oxidation (*P*_M_*) and PSII maximal quantum efficiency (*F*_V_/*F*_M_) from intact leaves with Dual‐KLAS‐NIR (Figure 2a,b). PSI photoinhibition was assigned moderate when around 60% of PSI complexes (*P*_M_*) were inhibited and recovery to 45% inhibition was recorded after 24 h in growth conditions. Severe PSI photoinhibition treatment of leaves resulted in around 85% inhibition, with a recovery to 60% inhibition during 24 h in growth conditions. Partial recovery of *P*_M_* indicated that plants can repair some of the damaged iron‐sulfur clusters of PSI via a still unidentified mechanism as discussed before (Tiwari et al., 2016). PSII maximal quantum efficiency (*F*_V_/*F*_M_) was only slightly affected by the moderate or severe PSI photoinhibition treatment and was partially restored during 1 day in growth conditions (Figure 2b). Leaf chlorophyll content and chlorophyll a/b ratio were not substantially changed by the inhibition treatment (Figure 2c,d).

### 3.2 Consequences of PSI photoinhibition to carbon assimilation and functionality of light reactions

Effects of PSI photoinhibition on CO_2_ assimilation and functional characteristics of photosynthetic light reactions were analysed from intact leaves with infra‐red gas analyser GSF‐3000. The gas analyser was connected to P700, PC, Fd and chlorophyll a fluorescence measuring system Dual‐KLAS‐NIR with a Dual‐PAM‐100 gas‐exchange cuvette for parallel measurement of all parameters.

Moderate and severe PSI photoinhibition altered differently the CO_2_ assimilation rate as a response to applied light intensity (Figure 2e and Supporting Information: Figure 1). Directly after PSI photoinhibition treatment, the dark respiration was enhanced, whereas no difference existed between control plants and plants that had recovered from the inhibition for 24 h in growth conditions (Figure 2e). Under low light illumination, the differences between moderately and severely inhibited plants were most striking, as the severely inhibited plants were still respiring without any capability for net assimilation of CO_2_ (Figure 2e). Partial restoration of CO_2_ assimilation was recorded in moderately inhibited and 24 h‐recovered plants (Figure 2e). PSI photoinhibition decreased the net CO_2_ assimilation by 40% in moderately and 65% in severely inhibited plants when measured under growth light illumination, whereas almost full recovery was recorded in moderately inhibited plants after 24 h recovery in normal growth conditions (Figure 2e). High light illumination abolished the distinct decline in CO_2_ assimilation rates of plants exposed to moderate and severe PSI photoinhibition treatments and resulted in similar CO_2_ assimilation rates for control, PSI photoinhibited and recovered plants (Figure 2e).

**Figure 3:**
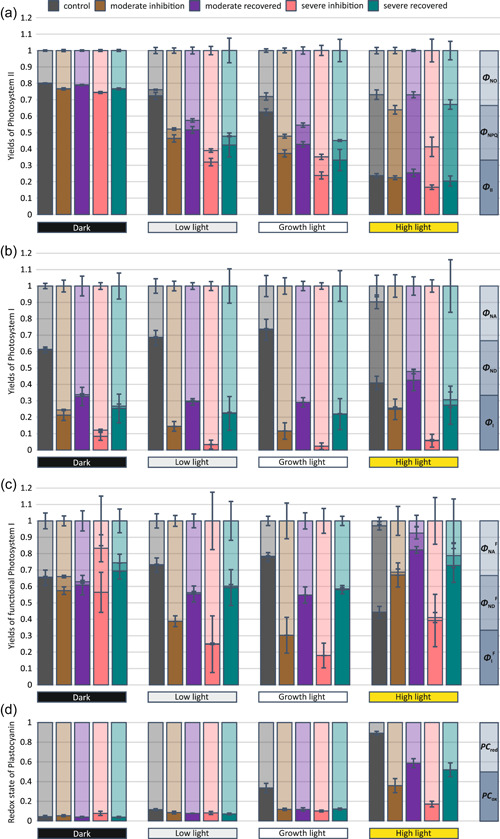
Photosynthetic parameters from plants exposed for 5 min to four different light intensities: dark, low light (35 µmol photons m^−2^ s^−1^), growth light (165 µmol photons m^−2^ s^−1^) and high light (635 µmol photons m^−2^ s^−1^). (a) Quantum yields of photosystem II: PSII photochemistry (Φ_II_), regulated energy dissipation (Φ_NPQ_) and nonregulated energy dissipation (Φ_NO_). The three yield values are stacked into each column: Φ_II_ at the bottom, Φ_NPQ_ in the middle and Φ_NO_ on the top with dark, moderate and light colours, respectively. (b) Quantum yields of photosystem I: PSI photochemistry (Φ_I_), donor side limitation (Φ_ND_), and acceptor side limitation (Φ_NA_). The three yield values are stacked into columns: Φ_I_ at the bottom, Φ_ND_ in the middle and Φ_NA_ at the top with dark, moderate and light colours, respectively. (c) Quantum yields of functional photosystem I: PSI photochemistry (Φ_I_^F^), donor side limitation (Φ_ND_^F^) and acceptor side limitation (Φ_NA_^F^). The three yield values are stacked into columns: Φ_I_^F^ in the bottom, Φ_ND_^F^ in the middle and Φ_NA_^F^ at the top with dark, moderate and light colours, respectively. (d) Steady‐state redox state of plastocyanin (PC): fractions of reduced PC (PC_red_) and oxidized PC (PC_ox_). Two values are stacked into columns: PC_red_ at the bottom and PC_ox_ at the top with dark and light colours, respectively. Parameters were measured from plants directly after PSI photoinhibition treatment and from plants that had recovered for 24 h in growth conditions after inhibition treatment. Control plants were taken directly from growth conditions. Measurements were done with saturating pulse method after a minimum of 30 min dark acclimation. Error bars show SDs among replicates (*n* ≥ 4). [Color figure can be viewed at wileyonlinelibrary.com]

As to the functionality of the light reactions, both moderate and severe PSI photoinhibition exerted a clear effect also on the performance of PSII (Figure 3a). The effect was most drastic under low and growth light illumination, as the photochemical yield of PSII (Φ_II_) was 40%–60% lower in moderately and severely inhibited plants compared to control plants, mostly due to a higher yield of nonregulated energy dissipation (Φ_NO_). The yield of regulated energy dissipation (Φ_NPQ_) was higher in PSI photoinhibited plants under low light illumination (Figure 3a and Supporting Information: Figure 2), which would imply that state‐transition induced quenching (qT) is higher in the inhibited plants compared to control plants, based on the fact that energy‐dependent quenching (qE) is not activated under low light illumination. On the other hand, no differences in Φ_NPQ_ were observed under growth light illumination (Figure 3a), where a partial induction of qE in control plants masked the impact of qT on total non‐photochemical exciton quenching (NPQ). Under high light illumination, Φ_II_ of inhibited plants did not generally differ from that of control plants, except for severely PSI‐inhibited plants measured directly after the inhibition treatment, which demonstrated lower Φ_II_ (Figure 3a). This was accompanied by twice the amount of Φ_NO_ and 50% lower Φ_NPQ_, in comparison with the control plants.

As expected, the moderate and severe PSI photoinhibition treatment reduced the photochemical yield of PSI (Φ_I_) (Figure 3b). This was most notable in plants under low and growth light illumination, where Φ_I_ was reduced due to a substantial increase in acceptor side limitation (Φ_NA_). The effect of PSI inhibition on Φ_I_ was somewhat smaller in plants under high light illumination and plants recovered for 24 h from moderate inhibition had already reached the control level. These high light effects were mostly due to a markedly lower donor side limitation (Φ_ND_) in inhibited and recovered plants, in comparison with the control plants.

To disclose changes in stromal electron acceptors of PSI, we calculated the yields of functional PSI complexes (denoted with upper case F; Figure 3c). In PSI photoinhibited plants, Φ_I_^F^ was lower under low and growth light illumination conditions compared with the control, whereas under high light illumination Φ_I_^F^ was equal to or higher than in the control. The Φ_NA_^F^ of PSI photoinhibited plants was still higher than that in the control plants. This was valid at all different light intensities, with darkness as the only exception. Plants that had recovered for 24 h in growth conditions showed lower Φ_NA_^F^ than plants analysed directly after photoinhibition treatment.

Consequences of PSI photoinhibition to intersystem electron transfer chain were monitored by recording the steady‐state redox state of PC (Figure 3d). High light illumination induced the most prominent changes in the reduction state of PC, increasing the reduced fraction of PC from six to eightfold in moderately and severely PSI photoinhibited plants as compared with control plants (Figure 3d). Although the plants measured after 24 h recovery in growth conditions had partially restored the oxidation state of the PC pool, it remained well below the respective value of control plants. Smaller changes in PC redox state were apparent under growth light illumination and practically no differences were evident between plants measured directly after PSI inhibition and after 24 h recovery. Darkness or low light illumination did not alter the PC redox state in PSI photoinhibited plants with respect to the control plants (Figure 3d).

### 3.3 24 h recovery from PSI photoinhibition treatment alters the distribution of electrons to carbon assimilation under growth light illumination

**Figure 4:**
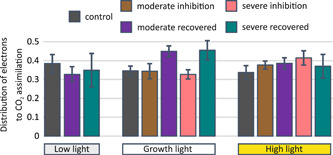
Distribution of reducing power to CO_2_ assimilation in plants exposed for 5 min to three different light intensities: low light (35 µmol photons m^−^^2^ s^−1^), growth light (165 µmol photons m^−2^ s^−1^) and high light (635 µmol photons m^−2^ s^−1^). CO_2_ assimilation and PSII electron transfer (ETRII) were measured from plants directly after Photosystem I (PSI) photoinhibition and from plants that had recovered for 24 h in growth conditions after inhibition. Control plants were taken directly from growth conditions. All plants were dark acclimated for a minimum of 30 min before measurements. Error bars show SDs among replicates (*n* ≥ 4). [Color figure can be viewed at wileyonlinelibrary.com]

CO_2_ assimilation appeared to recover faster from PSI photoinhibition than the function of PSII (Figures 2e and 3a). Therefore, we estimated the relative proportion of electrons allocated to CO_2_ assimilation (Figure 4), by calculating the ratio of CO_2_ assimilation to PSII electron transfer (ETRII). The plants measured directly after PSI photoinhibition exhibited high respiration rates under low light illumination (Figure 2e) and therefore we excluded these measurements from this inspection. When the 24 h‐recovered and control plants were illuminated with low light and high light, no differences in the ratio of CO_2_ assimilation to ETRII were detected (Figure 4). By contrast, the ratio was higher in the 24 h‐recovered plants than in control plants when illuminated at growth light intensity indicating that more reducing power is distributed to CO_2_ assimilation at the expense of other reductant consuming reactions. As the high phosphorylation of light‐harvesting complex II protein (LHCII) alters the distribution of excitation energy between the two photosystems, the ETRII value was corrected based on the 77 K fluorescence spectra (see below) and the distribution of excitation energy was recalculated (Supporting Information: Figure 3). Such correction further strengthened our initial interpretation of enhanced distribution of reductants to CO_2_ assimilation at the expense of other reductant‐consuming reactions, as the ETRII of inhibited plants was lower than that of the control plants.

### 3.4 Modulation of pmf and thylakoid proton conductivity by PSI photoinhibition

**Figure 5:**
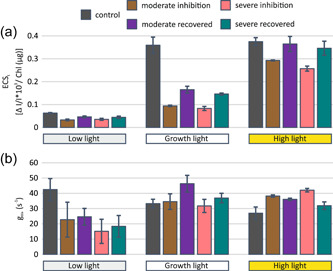
(a) ECS_t_ and (b) g_H+_ from plants exposed for 5 min to three different light intensities: low light (35 µmol photons m^−2^ s^−1^), growth light (165 µmol photons m^−2^ s^−1^) and high light (635 µmol photons m^−2^ s^−1^). Parameters were measured from plants directly after Photosystem I (PSI) photoinhibition and from plants that had recovered for 24 h in growth conditions after inhibition. Control plants were taken directly from growth conditions. Measurements were done with the dark interval relaxation kinetics method after a minimum of 30 min dark acclimation. ECS_t_ was normalized to the chlorophyll content of 10 mm diameter leaf discs cut from measured leaves. Error bars show SDs among replicates (*n* ≥ 3). [Color figure can be viewed at wileyonlinelibrary.com]

The effect of PSI photoinhibition on generation of light‐induced pmf (ECS_t_) (Figure 5a) and thylakoid membrane proton conductance (g_H+_) (Figure 5b) was investigated by monitoring the electrochromic shift with Dual‐PAM‐100 equipped with the P515/535 module, using the dark interval relaxation kinetics method. Under growth light illumination, PSI photoinhibition exerted a major effect on ECS_t_ (Figure 5a). The ECS_t_ of inhibited plants was only 25%–30% of that in the control plants and was restored only slightly during the 24 h recovery in growth conditions. Under low light illumination, the differences in ECS_t_ between the control and PSI photoinhibited plants were smaller; however, the trend was the same. Under high light illumination, PSI photoinhibition reduced ECS_t_ in plants monitored directly after the photoinhibition treatment, but ECS_t_ was almost completely restored in plants recovered for 24 h (Figure 5a). PSI photoinhibition exerted a distinct effect on g_H+_ in comparison to ECS_t_ (Figure 5b). Under low light illumination, g_H+_ was lower in inhibited than in control plants, but under growth light illumination the values were equal or greater than in the control. Moreover, under high light illumination, most of the inhibited plants exhibited higher g_H+_ than the control and the 24 h‐recovered plants had lower g_H+_ than those measured directly after PSI photoinhibition treatment.

### 3.5 PSI photoinhibition alters the stoichiometry of thylakoid protein complexes

**Figure 6:**
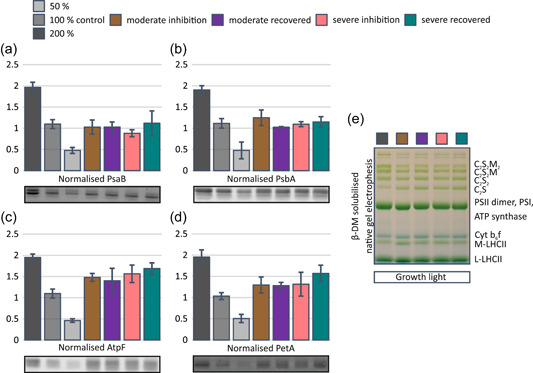
The effect of Photosystem I (PSI) photoinhibition on the abundance of major photosynthetic complexes. (a) Amount of PSI subunit PsaB. (b) Amount of PSII subunit PsbA. (c) Amount of ATP synthase subunit AtpF. (d) Amount of Cyt b_6_f subunit PetA. (e) β‐DM solubilized blue native gel electrophoresis of intact thylakoid complexes. Leaf material for thylakoid isolation was collected after 30 min dark treatment followed by 1 h treatment in growth light (165 µmol photons m^−2^ s^−1^). Plants were taken to the dark treatment directly after PSI inhibition and after 24 h recovery in growth conditions after inhibition. Control plants were taken to the dark treatment directly from growth conditions. SDS samples for subunit determination were loaded based on chlorophyll amount: 2, 1 and 0.5 µg for control dilution series and 1 µg for inhibited samples. Solubilized proteins were separated with sodium dodecyl‐sulfate polyacrylamide gel electrophoresis (SDS‐PAGE) and transferred to a polyvinylidene difluoride (PVDF) membrane. Proteins were immunodetected with specific antibodies against PsaB, PsbA, AtpF and PetA. Signals were normalized to a linear regression of signals of the control dilution series. Error bars show SDs among three biological replicates. For the determination of protein complexes, equal amounts of thylakoids (5 µg chlorophyll) were solubilized with β‐*D*‐dodecyl maltoside (β‐DM) and separated by blue native PAGE (BN‐PAGE). [Color figure can be viewed at wileyonlinelibrary.com]

To find out whether the functional differences in PSI photoinhibited plants result from changes in the stoichiometry of protein complexes in the thylakoid membrane, we determined the amounts of distinct protein subunits of major photosynthetic protein complexes. There was a slight decrease in the amount of PSI subunit PsaB in severely inhibited plants (Figure 6a), whereas the amount of the PSII subunit PsbA was not affected by the photoinhibition treatment (Figure 6b). The effect of PSI photoinhibition on ATP synthase subunit AtpF (Figure 6c) and Cyt b_6_f complex subunit PetA (Figure 6d) was more notable. AtpF amount increased during the inhibition treatment and remained at an elevated level during the subsequent 24 h recovery. A similar trend was observed for PetA, but the changes were smaller than those for AtpF.

As the amounts of ATP synthase and Cyt b_6_f complex subunits (AtpF and PetA) were affected in PSI photoinhibited plants, we next studied the composition of thylakoid protein complexes with blue native gel electrophoresis (BN‐PAGE), which maintains the photosynthetic protein complexes intact. As expected, the amount of Cyt b_6_f was increased in PSI photoinhibited plants (Figure 6e). ATP synthase, unfortunately, co‐migrates with the PSII dimer (slightly lower) and therefore could not be assessed from the BN‐gels.

In addition to changes in the amounts of major complexes, PSI photoinhibition treatment altered the composition of PSII–LHCII supercomplexes and the PSI‐inhibited samples showed reduced amounts of large supercomplexes (C_2_S_2_M_2_ and C_2_S_2_M) (Figure 6e). This was accompanied by an increase in the free moderately bound M‐LHCII, which comprises LHCII trimers associated with tightly bound minor light‐harvesting antennae proteins, Lhcb4 and Lhcb6. Conversely, the amount of loosely bound L‐LHCII was not noticeably affected by the PSI photoinhibition treatment.

### 3.6 Plasticity and dynamics of thylakoid protein complexes upon PSI photoinhibition

**Figure 7:**
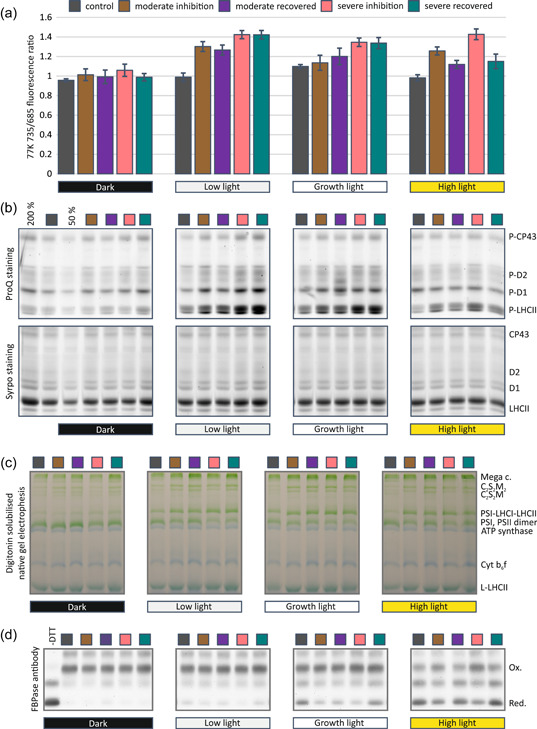
Effect of Photosystem I (PSI) photoinhibition on dynamic regulation of thylakoid protein complexes and the redox state of the stromal enzyme fructose 1,6‐bisphosphatase. Control plants, PSI photoinhibited and 24 h‐recovered plants were exposed to darkness for 30 min before 1 h treatments under the four different light intensities: dark, low light (35 µmol photons m^−2^ s^−1^), growth light (165 µmol photons m^−2^ s^−1^) or high light (635 µmol photons m^−2^ s^−1^). (a) Distribution of excitation energy between the two photosystems, expressed as F735/F685 ratio. 77 K fluorescence emission spectra were recorded from isolated thylakoids which were diluted into 10 µg chlorophyll/1 ml. Fluorescence spectra were measured with 480 nm excitation light in liquid nitrogen. (b) Phosphorylation of thylakoid proteins. Thylakoid samples were loaded based on chlorophyll amount and proteins were separated with sodium dodecyl‐sulfate polyacrylamide gel electrophoresis (SDS‐PAGE). Separated proteins were first stained with ProQ and imaged, followed by staining with Sypro and imaging. (c) Analysis of native thylakoid protein complexes. Thylakoid membranes were solubilized with digitonin and the solubilized protein complexes were separated with blue native PAGE (BN‐PAGE). (d) Reduced (Red) and oxidized (Ox) form of Fructose 1,6‐bisphosphatase (FBPase) determined with redox labelling. After the light treatments leaf samples were immediately frozen in liquid nitrogen, followed by isolation of proteins. Protein thiols were blocked with N‐ethyl‐maleimide followed by the reduction of residual protein disulfides with dithiothreitol (DTT) and labelling of exposed thiols with pegylated maleimide that increases the apparent molecular mass of the protein by 5 kDa per thiol group. Labelled proteins were separated with SDS‐PAGE and transferred to a polyvinylidene difluoride (PVDF) membrane. Amounts of differentially labelled proteins were determined with specific antibody. [Color figure can be viewed at wileyonlinelibrary.com]

As shown above, PSI photoinhibition altered the formation of PSII–LHCII supercomplexes (Figure 6e), which prompted us to check whether this change in the structural organization of light‐harvesting is reflected at the functional level. To this end, we isolated thylakoids from PSI photoinhibited and control plants illuminated for 1 h in the four different light intensities, which were also applied in functional studies of intact leaves (Figure 1). The distribution of excitation energy between PSII and PSI was addressed by recording the low‐temperature fluorescence emission spectra from highly diluted suspensions of isolated thylakoids. PSI inhibition did not alter the peak positions of the 77 K fluorescence spectra, which implied that the light‐harvesting antennae remained connected to the reaction centres (Supporting Information: Figure 4). However, the ratio of fluorescence emitted from PSII (peaking at 685 and 695 nm) to that emitted from PSI (peaking at 735 nm) was affected by PSI photoinhibition, but the difference was highly dependent on preillumination light intensity (Figure 7a). Although the effect was minor in darkness, the low light and growth light illumination for 1 h enhanced the excitation of PSI, with no restoration during the 24 h recovery period. After 1 h preillumination of plants in high light, the situation was different and the excitation energy distribution between PSII and PSI was closer to that in control plants.

To get further insights into the redox conditions that drive the dynamics of excitation energy distribution between PSII and PSI in the thylakoid membrane, we next analysed how the phosphorylation levels of the LHCII and PSII core proteins, indicative of the redox state of the PQ‐pool, were affected by the four different light treatments of the control, PSI photoinhibited and subsequently 24 h‐recovered plants. Thylakoid proteins isolated from PSI‐inhibited and control plants were separated with SDS‐PAGE and stained with a phosphoprotein‐specific stain. Total protein stain was used to control the equal loading of the samples (Figure 7b). PSI photoinhibition treatments increased the phosphorylation level of LHCII proteins in all 1 h light treatments, except for darkness, in comparison with the control plants. Phosphorylation of PSII core proteins (D1, D2 and CP43) in low and growth light was also higher in PSI‐inhibited plants compared with that in the control. On the contrary, there were no major differences in PSII core protein phosphorylation between the samples illuminated at high light, except for a slight decrease that was observed in 24 h‐recovered plants as compared with the control plants. The phosphorylation of LHCII proteins was, in general, weaker in high light in comparison with the low and growth light‐treated plants, but still higher in PSI inhibited plants compared with the control. Moreover, the plants that recovered for 24h in growth conditions had lower LHCII phosphorylation after 1 h exposure to high light, in comparison with the plants analysed immediately after inhibition.

Dynamics of LHCII protein phosphorylation and concomitant changes in excitation energy distribution between PSII and PSI have been attributed to reversible formation and disassembly of the PSI–LHCI–LHCII supercomplex, regulated via LHCII phosphorylation by the state transition 7 (STN7) kinase (Pesaresi et al., 2009). Therefore, the PSI–LHCI–LHCII supercomplex was visualized from thylakoids after 1 h differential light treatments of plants, using digitonin for solubilizing the protein complexes followed by separation of the complexes with BN‐PAGE (Figure 7c). PSI photoinhibition increased the amount of PSI–LHCI–LHCII supercomplexes in all other light conditions, except darkness. The effect was most striking in high light‐treated plants, where the control demonstrated only a minor amount of PSI–LHCI–LHCII supercomplex, but the inhibited and 24 h‐recovered plants showed similar amounts to those of the growth and low light samples. It also appeared that the PSI photoinhibition treatment led to a higher amount of digitonin solubilized PSII–LHCII supercomplexes (C_2_S_2_M_2_ and C_2_S_2_M) and Cyt b_6_f compared with the control in all other light conditions except darkness.

### 3.7 Redox regulation of stromal metabolism is affected by PSI inhibition

Sustenance of the LHCII phosphorylation and consequently the PSI–LHCI–LHCII supercomplex in PSI photoinhibited plants under high light illumination suggested modulations in stromal redox conditions in comparison to control plants. This was further investigated by determining the amount of reduced and oxidized forms of FBPase from control and PSI photoinhibited plants subjected to the four different light conditions (Figure 7d). Photoinhibition treatment, moderate or severe, diminished the level of reduced FBPase in all light conditions. Nevertheless, reduced FBPase was restored to control levels in 24 h‐recovered plants.

## 4 DISCUSSION

The photosynthetic apparatus is harnessed with mechanisms that continuously control the excitation energy distribution to and between the two photosystems to keep ETC optimally oxidized (Bassi & Dall'Osto, 2021; Tikkanen & Aro, 2014). ETC oxidation, on the other hand, is highly dependent on the capacity of the CBB cycle, which functions as the main electron sink from ETC. CBB cycle is likewise impacted by different biotic and abiotic factors, which heavily modify CO_2_ availability and the rate of chemical reactions. Keeping the ETC optimally oxidized is important, as any condition that results in the accumulation of excess electrons in ETC, such as an abrupt increase in light intensity, easily leads to PSI photoinhibition (Grieco et al., 2012). The sensitivity of PSI to photoinhibition under high or fluctuating light is dependent on plant species (Huang et al., 2018; Terashima et al., 2021; Yamori et al., 2016). Moreover mutants, with problems in regulation of electron transfer, particularly the PGR5 deficient mutants, are especially prone to PSI photoinhibition (Grieco et al., 2012; Suorsa et al., 2012; Yamori et al., 2016). PSI shows extremely slow recovery capacity from photoinhibition, both when induced by illumination of plants at low temperature (Kudoh & Sonoike, 2002; Zhang & Scheller, 2004) and by a mere high or fluctuating light treatment (Lima‐Melo et al., 2019; Suorsa et al., 2012), when compared with a much higher recovery capacity of plants from PSII photoinhibition. The time course of recovery at growth conditions from low‐temperature PSI photoinhibition (Kudoh & Sonoike, 2002; Zhang & Scheller, 2004) and our targeted PSI photoinhibition treatment also differ from each other, being much longer after the PSI inhibition occurring at low temperature and thus being mixed with cold acclimation responses. Notably, no degradation of PSI proteins and chlorophylls were recorded upon the recovery period of 24 h from targeted PSI photoinhibition treatment (Figures 2c and 6a). Although the design of our PSI inhibition treatment (Tikkanen & Grebe, 2018) does not directly mimic any natural light condition, it provides a unique opportunity to disclose the effects of specific inhibition of only the PSI centers on thylakoid redox homoeostasis and elucidation of subsequent mechanisms activated for restoration of chloroplast redox balance, an important component of plant's acclimation strategy.

Considering how the plants cope with accumulation of damaged PSI centres, it is likely that mechanisms capable of mitigating the consequences of PSI deficiency are activated directly after the damage. To disclose such acclimation mechanisms, we subjected *Arabidopsis* plants to specific photoinhibition treatments at normal growth temperature, which reduced the number of functional PSI centres by 60% (moderately photoinhibited) or 85% (severely photoinhibited) (Figure 2a). The properties of the PSI‐depleted photosynthetic machinery were subsequently investigated both directly after the PSI inhibition treatment and after a 24 h ‘recovery’ period in growth conditions.

### 4.1 Restoration of the CO_2_ assimilation capacity after PSI photoinhibition is not dependent on PSI recovery

It was of primary importance to understand how the reduced number of functional PSI centres (Figure 2a) impacts the CO_2_ assimilation rate in different light intensities and whether the subsequent acclimation of plants to the PSI‐limited state during the 24 h recovery period changes the relationship between the abundance of functional PSI and CO_2_ assimilation rate (Figure 2a,e). Our results demonstrate that the decrease in the abundance of functional PSI, recorded immediately after the PSI photoinhibition treatment, slowed down the CO_2_ assimilation rate only when measurements were conducted under low and growth light intensities (Figure 2e). On the contrary, under higher light intensity the difference in CO_2_ assimilation rate between the PSI photoinhibited and control plants strongly diminished (Figure 2e). These results imply that the plants make use of only a small fraction of their entire PSI pool to reach a maximal CO_2_ assimilation rate in high light conditions. This might allow plants to lower the PSI to PSII ratio as generally seen during long‐term high light acclimation (Bailey et al., 2001; Flannery et al., 2021). Nonetheless, during the 24 h recovery period after PSI photoinhibition treatment, the CO_2_ assimilation rates which were recorded under low and growth light illumination increased from that recorded immediately after the photoinhibition treatment (Figure 2e). These results demonstrate that the 24 h recovery period after the PSI inhibition treatment, while not long enough for major PSI repair, nevertheless provides the means for the photosynthetic machinery to partially restore the CO_2_ assimilation rate also under low and moderate light intensities. In addition to these effects, it is likely that the excitation energy distribution to photoinhibited PSI centres decreases and conversely the energy transfer to functional PSI centres increases. Although we could not obtain experimental evidence of this process, it is something that must occur at the latest when the inhibited PSI centres are being degraded. Thus, this process could partially explain the observed increase in CO_2_ assimilation rate in light‐limited conditions upon recovery for 24 h in growth conditions (Figure 2e).

### 4.2 PSI photoinhibition limits LET

PSI photoinhibition has two main consequences on light reactions. Firstly, the damaged and therefore permanently acceptor side limited PSI centres still receive excitation energy, which hinders the availability of light for functional photosystems (Zivcak et al., 2015). This aggravates the light limitation of photosynthesis, especially at low light intensity (Figures 3b and 2e). Secondly, severe damage of PSI, in connection with only minor inhibition of PSII, results in a high reduction state of the PC pool (Figure 3d) and, more importantly, of the PQ pool, which leads to acceptor side limitation at PSII, seen as low qP (Supporting Information: Figure 5). Such a decrease in the proportion of open PSII reaction centres is reflected as an increase in nonregulated energy dissipation (Φ_NO_) and as a lower photochemical yield of PSII (Φ_II_) (Figure 3a).

### 4.3 Changes in redox regulation allow restoration of CO_2_ assimilation without major PSI repair

An increase in the distribution of light energy to PSI (Figure 7a and Supporting Information: Figure 4) implies thylakoid protein phosphorylation as a potential molecular mechanism behind the restoration of functional balance between photosystems in PSI photoinhibited leaves. Phosphorylation of both the LHCII and PSII core proteins catalysed by the STN7 and STN8 kinases, respectively (Bellafiore et al., 2005; Bonardi et al., 2005), was substantially enhanced in PSI photoinhibited leaves (Figure 7b). Both kinases are redox‐regulated and principally activated by the reduction of the PQ pool. STN7 kinase is additionally regulated by the stromal Trx system (Rintamäki et al., 1997, 2000), being inhibited by reduced Trx in conditions of excess light (Ancín et al., 2019). Nevertheless, the stromal redox network of PSI photoinhibited plants was maintained fairly oxidized (Figure 7d), contrary to that of the PQ pool, thereby favouring high STN7 kinase activity and LHCII phosphorylation in the thylakoid membrane throughout the different light conditions, also under high light illumination (Figure 7b). The high level of LHCII phosphorylation leads to formation of the PSI–LHCI–LHCII complex (Figure 7c), which allows PSI to receive more excitation energy from the LHCII‐lake (Benson et al., 2015; Grieco et al., 2015; Schiphorst et al., 2021). PSII core phosphorylation, on the other hand, destabilizes the larger C_2_S_2_M_2_ and C_2_S_2_M PSII supercomplexes (Figure 6e), lowering the excitation energy partitioning from LHCII‐lake to PSII reaction centres (Dietzel et al., 2011). Both of these changes in protein complex interactions act synergistically to increase the relative excitation of PSI, which endeavours to balance the activity of the photosystems in PSI photoinhibited plants. This is demonstrated as an increase in the share of excitation energy transfer from LHCII to PSI in all light conditions except darkness (Figure 7a).

The imbalance between the abundance of functional photosystems generated by the PSI photoinhibition treatment of plants is extremely high (Figure 2a,b) and in light‐limited conditions, the phosphorylation‐dependent mechanisms do not get completed in the timescale of the measurement to restore the balance between the two photosystems in its full capacity, seen as a high yield of nonregulated energy dissipation (Φ_NO_) (Figure 3a). Upon high light illumination, the situation is different and the remaining functional PSI centres receive sufficient excitation energy and increase the photochemical yield of functional PSI (Φ_I_^F^) to a higher level than measured for control plants (Figure 3c). On the other hand, in plants measured directly after the PSI photoinhibition treatment, the yield of nonregulated energy dissipation (Φ_NO_) remained higher than in control plants. However, at the same time, the yield of regulated energy dissipation (Φ_NPQ_) was lower than in control plants, which allowed the plants to reach similar levels of the photochemical yield of PSII (Φ_II_) as the control leaves (Figure 3a). These alterations in excitation energy distribution and dissipation allowed the PSI photoinhibited plants to reach the CO_2_ assimilation values closer to those in control plants under high light illumination (Figure 2e).

The reducing capacity of the stromal Trx system was restored to control levels during the 24 h recovery period after PSI photoinhibition (Figure 7d), although the amount of functional PSI did not recover completely (Figure 2a). Restoration of Trx system activity was reflected in an increase in the relative amount of reduced FBPase in light (Figure 7d), which can be seen as lower Φ_NA_^F^ (Figure 3c). Restoration of the Trx system was also seen in the activity of STN7 kinase, demonstrated by a lowered phosphorylation level of LHCII in high light‐illuminated leaves (Figure 7b), which in turn was reflected in excitation energy distribution (Figure 7a). This data implies that the capacity of light reactions to reduce these stromal components increased during the 24 h recovery period.

Directly after PSI photoinhibition, the CO_2_ assimilation and photochemical yield of PSII (Φ_II_) decreased in synchrony (Figures 2e and 3a). However, the situation was changed after the 24 h recovery period and, especially under growth light illumination, the CO_2_ assimilation recovered more than the photochemical yield of PSII (Φ_II_). Such a large discrepancy between the photochemical yield of PSII (Φ_II_) and the CO_2_ assimilation rate can only be explained by the CBB cycle being favoured over other electron sinks downstream from ETC (Figure 4). Under growth light illumination, NA and malate valve comprise other probable sinks for reducing power. However, Fd has a lower affinity for nitrite reductase than for FNR, which implies that nitrite reduction functions efficiently only when Fd supply exceeds its consumption in NADPH formation (Baysdorfer & Robinson, 1985; Rachmilevitch et al., 2004). In addition, the chloroplastic malate dehydrogenase, functioning in the malate valve, is efficiently activated only when excess NADPH accumulates (Selinski & Scheibe, 2018). Therefore, it is conceivable that the CBB cycle is favoured over nitrite reduction and/or malate valve in these conditions since the sink capacity of the CBB cycle is increased.

### 4.4 PSI inhibition‐related increase in ATP synthase and Cyt b_6_f amounts modulates the regulation of light reactions

As a response to PSI photoinhibition, an increase in the abundance of ATP synthase and Cyt b_6_f took place and the elevated levels were maintained during the subsequent 24 h recovery period (Figures 6c–e). The factors controlling the stoichiometric biosynthesis of Cyt b_6_f and ATP synthase are poorly understood (Schöttler et al., 2015), yet it is clear that Cyt b_6_f, ATP synthase and the enzymes of the CBB cycle are tightly coregulated to maintain the balance between light reactions and stromal metabolism (Schöttler & Tóth, 2014; Vanlerberghe et al., 2019; Yamori et al., 2010). It is therefore likely that PSI photoinhibition‐induced imbalance in ETC initiates a redox cascade to upregulate the synthesis of these protein complexes. This is in line with previous observations showing that chronic high reduction state of the PQ pool in *stn7* mutant and PSI‐inhibited *pgr5* mutant induces an accumulation of ATP synthase (Suorsa et al., 2012; Tikkanen et al., 2006).

The elevated abundance of ATP synthase (Figure 6c) was reflected as an increase in thylakoid proton conductivity (g_H+_) (Figure 5b), although the contribution by several ion channels and transporters (Armbruster et al., 2017; Spetea et al., 2017) cannot be neglected. Nevertheless, the higher g_H+_ lowers light‐induced pmf (ECS_t_) and thus decreases the photosynthetic control and non‐photochemical quenching of excitation energy (Figures 5a and 3a,d). Consequently, the electron flow through Cyt b_6_f is enhanced not only by the increase in the abundance of the complex but also by a decrease in photosynthetic control. These modulations in Cyt b_6_f increase the reduction state of PC (Figure 3d) and decrease PSI donor side limitation (Figure 3b), which collectively allow the remaining undamaged PSI centres to function more efficiently (Figure 3c). It is evident that the increase in Cyt b_6_f and ATP synthase improves the capacity of the photosynthetic apparatus when the abundance of functional PSI is strongly reduced.

Noteworthy, even though the abundance of ATP synthase was maintained at a high level also during the 24 h recovery period (Figure 6c), g_H+_ returned close to that of control plants (Figure 5b). This provides evidence that upon the recovery period plants restore the capability to tune the activity of the ATP synthase. The Trx system contributes to the regulation of proton conductivity, which declines if the thiol‐redox state of chloroplast rises (Nikkanen et al., 2018). The lack of PGR5 protein is known to abolish the Trx‐dependent control of proton conductivity, suggesting that this phenomenon is mediated by PGR5 (Avenson et al. 2005; Nikkanen et al., 2018). Thus, the re‐establishment of the thiol‐redox state during the recovery period (Figure 7d) likely contributes to the restoration of control‐type proton conductivity in PSI photoinhibited plants, restoring the photosynthetic control and non‐photochemical quenching (Figure 3a,d).

### 4.5 Concluding remarks

**Figure 8:**
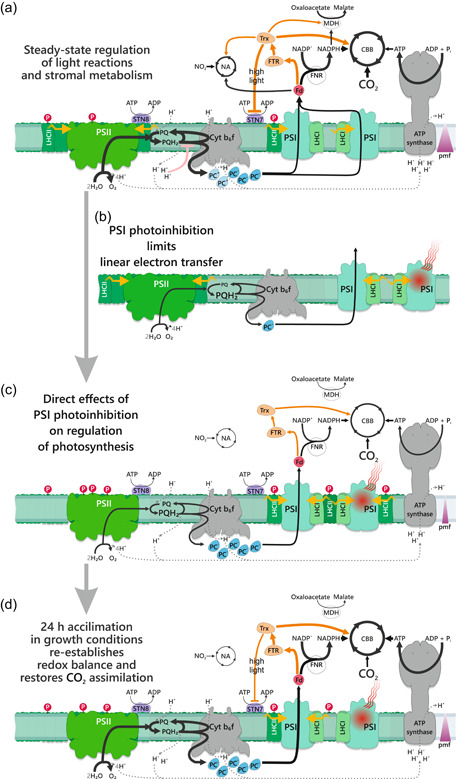
Scheme of Photosystem I (PSI) photoinhibition and subsequent induction of regulatory mechanisms postulated to allow PSI‐deficient plants to restore CO_2_ assimilation. (a) Balanced function of photosynthetic light reactions and stromal metabolism under steady‐state environmental conditions. Photosynthetic control, and the phosphorylation of light‐harvesting complex II (LHCII) and PSII core proteins, are modestly activated and reducing power from light reactions is fluently allocated to CO_2_ assimilation and other stromal sinks. (b) PSI photoinhibition limits linear electron transfer (LET). Damaged PSI reaction centres quench the excitation energy, and the plastoquinone (PQ) pool gets reduced limiting PSII activity. (c) PSI photoinhibition exerts direct effects on regulation mechanisms of photosynthesis. Firstly, PSI deficiency restricts electron flow to stromal acceptors and key reducing enzymes activating the Calvin–Benson–Bassham (CBB) cycle and other stromal components. Secondly, relaxation of the proton gradient (pmf) diminishes photosynthetic control at cytochrome b_6_f complex (Cyt b_6_f), facilitating electron flow to the plastocyanin (PC) pool. Thirdly, the reduction of the PQ pool further enhances the activity of Stn7 and Stn8 kinases (phosphorylate the LHCII and PSII core proteins, respectively) leading to the reorganization of the light‐harvesting antenna system to favour excitation of PSI. Notably, the canonical inhibition of LHCII phosphorylation in high light is prevented due to the lack of reduced Trx in the stroma. (d) 24 h acclimation to PSI‐deficiency results in restoration of CO_2_ assimilation. Enhanced function of PSI centres restores the activity of linear electron transfer (LET). This also restores the balanced redox state of the PQ pool and increases the photochemical yield of PSII. An increase in the amounts of Cyt b_6_f and ATP synthase, in turn, keeps the PC pool reduced and lowers the donor side limitation of PSI, thereby improving the photochemical yield of PSI. Concomitant changes in stromal regulation modulate the distribution of reductants between stromal sinks favouring the CBB cycle over other stromal sinks. The thickness of the black and orange lines represents the rate of reactions in LET and stromal metabolism. [Color figure can be viewed at wileyonlinelibrary.com]

Figure 8 collects together the results obtained from current experiments concerning PSI photoinhibition and subsequent redox‐driven modulations in regulation and acclimation of the photosynthetic apparatus to PSI deficient conditions. PSI is prone to photoinhibition in adverse environmental conditions when the imbalance between light reactions and stromal metabolism leads to the over‐reduction of PSI electron acceptors (Figure 8a,b). Recovery from PSI photoinhibition is an extremely slow process and thus PSI inhibition causes a long‐term reduction in the efficiency of the photosynthetic machinery. Here we demonstrate that plants circumvent the depletion of functional PSI by utilizing the acclimation mechanisms traditionally connected to short‐term and long‐term light acclimation. Initially, PSI inhibition reduces the electron flux via PSI, which lowers the formation of the pmf, reduces the PQ pool and oxidizes the stromal Trx‐system (Figure 8c). This induces acclimatory changes in the partitioning of excitation energy between PSII and PSI, and in the regulation of stromal metabolism. These changes reprogram the feedback regulation of light reactions. Besides such dynamic regulatory responses, also long‐term responses are induced leading to changes in the stoichiometry of the photosynthetic machinery (Figure 8d). Both the abundance and the activity of ATP synthase and Cyt b_6_f complex are increased and those changes play a central role in the regulation of photosynthesis and mitigation of PSI photoinhibition.

Our results highlight that the regulatory plasticity of the photosynthetic machinery provides the capacity to acclimate not only to changes in light conditions but also provides the ability to acclimate to any factor disturbing the homoeostasis of the electron transfer chain. We conclude that the light acclimation is composed not only of the responses to changes in light conditions but also of the mechanism mitigating the consequences of photoinhibition.

## Supporting information

Supporting information.
