# Supplementary material for: Plants acclimate to Photosystem I photoinhibition by readjusting the photosynthetic machinery
Source: Plant Cell Environ. 2022 Aug 16;45(10):2954–71. doi: 10.1111/pce.14400 (PMC9546127; doi:10.1111/pce.14400)
Supplement: Supplementary file 1 — Supporting information. [file PCE-45-2954-s001.docx]

# SUPPORTING INFORMATION

# Plants acclimate to Photosystem I photoinhibition by readjusting the photosynthetic machinery

Tapio Lempiäinen, Eevi Rintamäki, Eva-Mari Aro*, Mikko Tikkanen*

*Corresponding authors

# Molecular Plant Biology, Department of Life Technologies, University of Turku, Turku, Finland

**
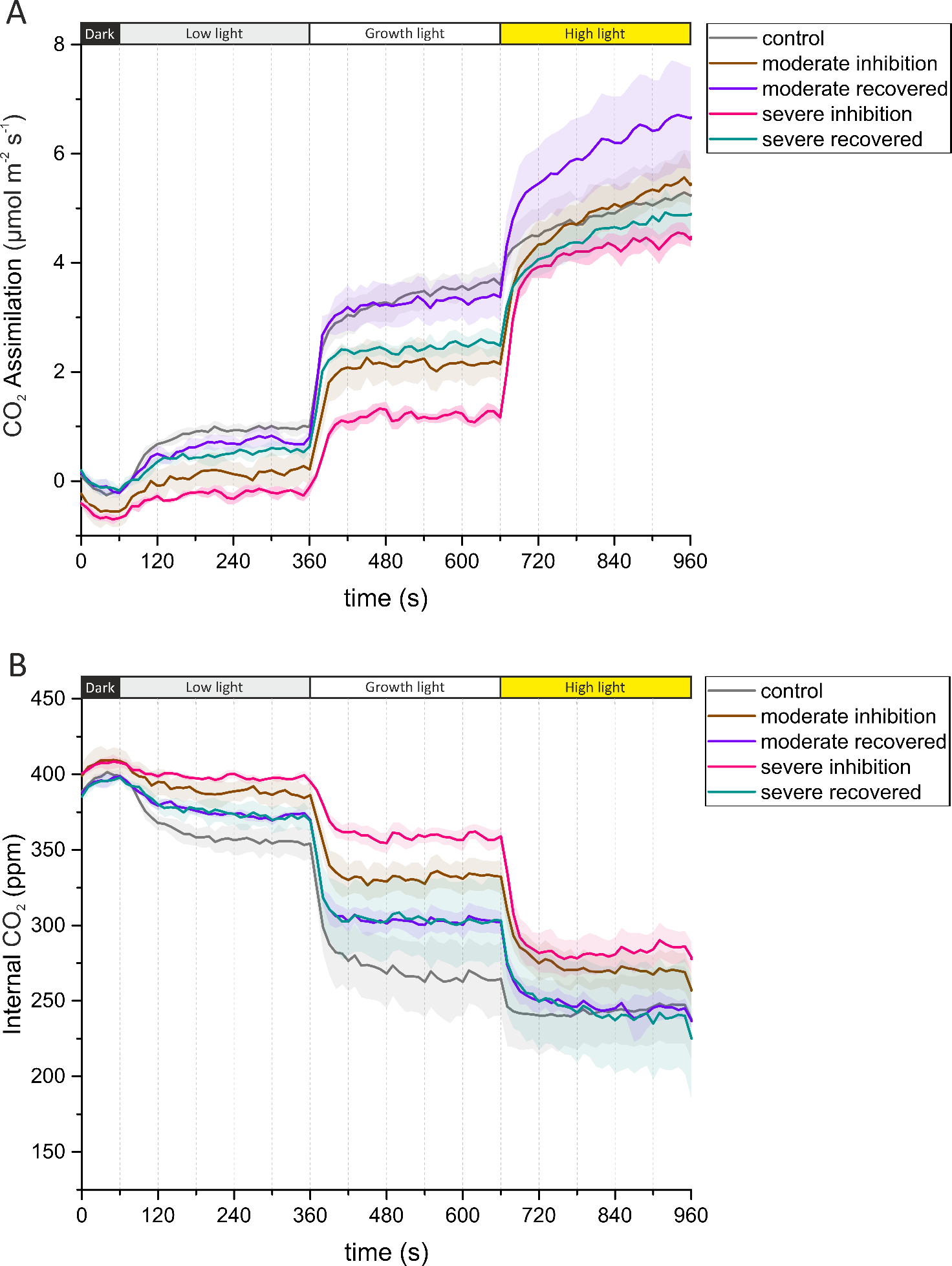
**

**Supplemental figure 1.** **A)** CO_2_ assimilation rates and **B)** internal CO_2_ concentrations of PSI photoinhibited and recovered plants recorded from four different light intensities: dark, low light (35 µmol photons m^-2^ s^-1^), growth light (165 µmol photons m^-2^ s^-1^) and high light (635 µmol photons m^-2^ s^-1^). Assimilation and internal CO_2_ concentration were measured from plants directly after PSI inhibition (moderate and severe inhibition) and from plants that had recovered for 24 h in growth conditions after inhibition (moderate and severe recovered). Control plants were taken directly from growth conditions (control). All plants were dark acclimated for a minimum of 30 min before subsequent illumination at the indicated light intensities for 5 min. Shaded areas show standard deviations among replicates (n ≥ 4). Internal CO_2_ concentration was calculated according to (von Caemmerer & Farquhar, 1981).


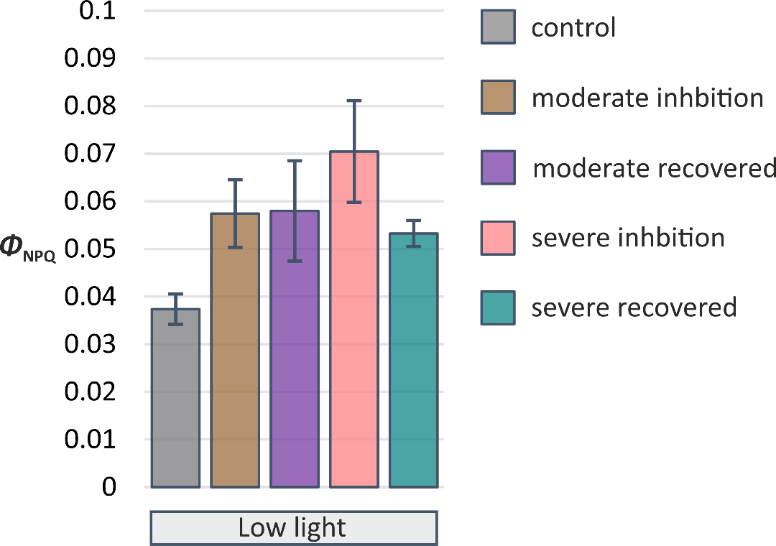


**Supplemental figure 2.** Quantum yields of regulated energy dissipation (*Φ*_NPQ_) from plants exposed for 5 min to low light (35 µmol photons m^-2^ s^-1^). *Φ*_NPQ_ was measured from plants directly after PSI photoinhibition treatment and from plants that had recovered for 24 h in growth conditions after inhibition treatment. Control plants were taken directly from growth conditions. Measurements were done with saturating pulse method after a minimum of 30 min dark acclimation. Error bars show standard deviations among replicates (n ≥ 4).

**
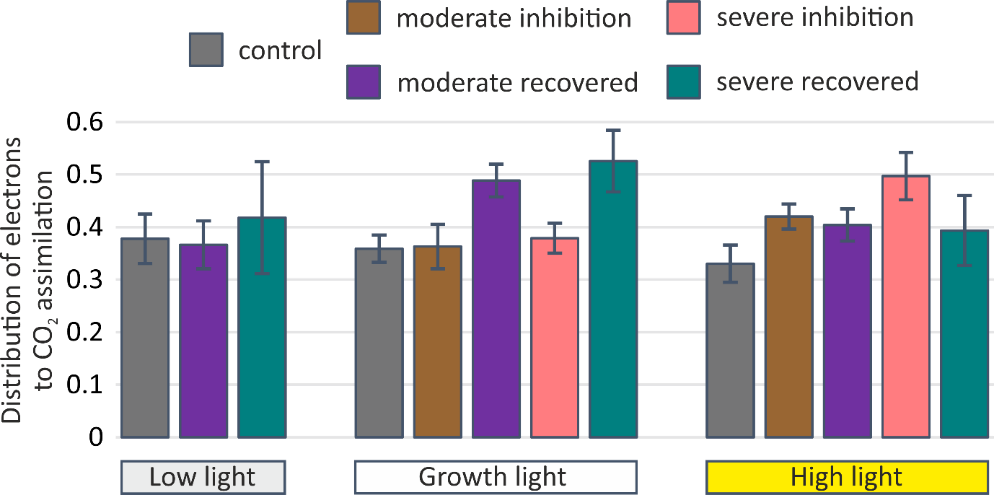
**

**Supplemental figure 3.** Distribution of reducing power to CO_2_ assimilation in plants exposed for 5 min to three different light intensities: low light (35 µmol photons m^-2^ s^-1^), growth light (165 µmol photons m^-2^ s^-1^) and high light (635 µmol photons m^-2^ s^-1^). CO_2_ assimilation and ETRII were measured from plants directly after PSI photoinhibition and from plants that had recovered for 24 h in growth conditions after inhibition. Light distribution between photosystem for ETRII calculations was determined from 77 K spectra. Control plants were taken directly from growth conditions. All plants were dark acclimated for a minimum of 30 min before measurements. Error bars show standard deviations among replicates (n ≥4).

**
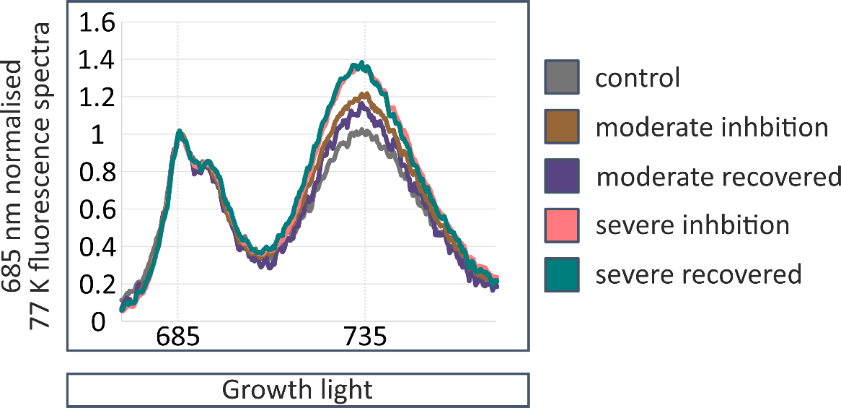
**

**Supplemental figure 4.** 77 K fluorescence spectra measured from thylakoids isolated from plants treated for 1 h under growth light illumination (165 µmol photons m^-2^ s^-1^). Isolated thylakoids were diluted into 10 µg chlorophyll / 1 ml and fluorescence spectra were recorded in liquid nitrogen using 480 nm excitation light. Fluorescence was normalised to 685 nm. Averages of three technical replicates are shown.

**
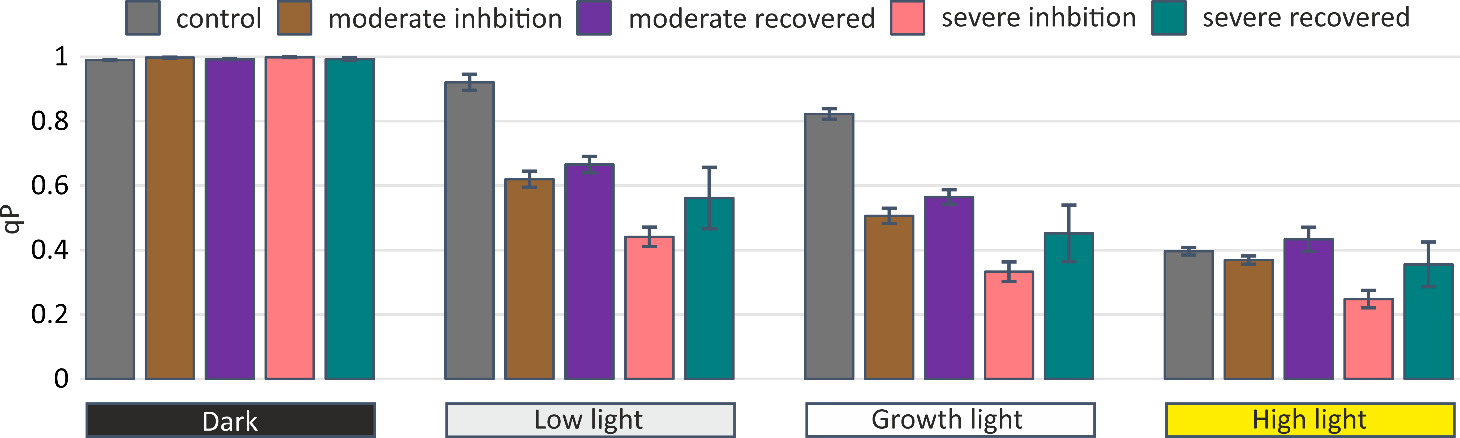
**

**Supplemental figure 5.** qP from plants exposed for 5 min to four different light intensities: dark, low light (35 µmol photons m^-2^ s^-1^), growth light (165 µmol photons m^-2^ s^-1^) and high light (635 µmol photons m^-2^ s^-1^). qP was measured from plants directly after PSI photoinhibition treatment and from plants that had recovered for 24 h in growth conditions after inhibition treatment. Control plants were taken directly from growth conditions. Measurement were done with saturating pulse method after a minimum of 30 min dark acclimation. Error bars show standard deviations among replicates (n ≥ 4). qP was calculated according to (Oxborough & Baker, 1997).

**References**

Oxborough, K., & Baker, N. R. (1997). Resolving chlorophyll a fluorescence images of photosynthetic efficiency into photochemical and non-photochemical components - Calculation of qP and Fv’/Fm’ without measuring Fo’. *Photosynthesis Research*, *54*(2), 135–142. https://doi.org/10.1023/A:1005936823310

von Caemmerer, S., & Farquhar, G. D. (1981). Some relationships between the biochemistry of photosynthesis and the gas exchange of leaves. *Planta*, *153*(4), 376–387. https://doi.org/10.1007/BF00384257
